# Effects and Limitations of a Unique, Nationwide, Self-Exclusion Service for Gambling Disorder and Its Self-Perceived Effects and Harms in Gamblers: Protocol for a Qualitative Interview Study

**DOI:** 10.2196/47528

**Published:** 2023-11-14

**Authors:** Anders Håkansson, Johanna Tjernberg, Helena Hansson

**Affiliations:** 1 Department of Clinical Sciences Lund, Psychiatry, Faculty of Medicine Lund University Lund Sweden; 2 Malmö Addiction Center, Gambling Disorder Unit Region Skåne, Competence Center Addiction Malmö Sweden; 3 Department of Psychiatry Region Skåne Lund Sweden; 4 School of Social Work Lund University Lund Sweden

**Keywords:** gambling disorder, problem gambling, behavioral addiction, self-exclusion, Spelpaus, gambling, management, data, online gambling, harm, harm reduction tool, recruitment, qualitative interview study, gambler

## Abstract

**Background:**

Voluntary self-exclusion from gambling is a common but underdeveloped harm reduction tool in the management of gambling problems or gambling disorders. Large-scale, multi-operator, and operator-independent self-exclusion services are needed. A recent nationwide multi-operator self-exclusion service in Sweden (Spelpaus), involving both land- and web-based gambling sites, is promising, but recent data have revealed limitations to this system and possibilities to breach one’s self-exclusion through overseas web-based gambling. More knowledge is needed about the benefits and challenges of such an extensive self-exclusion service, and its effects as perceived by gamblers.

**Objective:**

This study protocol describes the rationale and design of a qualitative interview study addressing the effects and limitations perceived by individuals with gambling problems and their concerned significant others. The study aims to provide an in-depth experience of this novel self-exclusion service and to inform stakeholders and policymakers in order to further improve harm reduction tools against gambling problems.

**Methods:**

Individuals with gambling problems will be recruited primarily through social media and also from a treatment unit, if needed, for a qualitative interview study. Recorded interview material will be analyzed through content analysis, and recruitment will continue until saturation in the material is reached. This study will provide in-depth information about a harm reduction tool that is promising and commonly used, but which has proven to be breached by a significant number of users, potentially limiting its efficiency. The aim is to interview a sufficient number of gamblers until saturation has been obtained in the interview material. Saturation will be considered through a continuous analysis, comparing recently collected data to previously collected data.

**Results:**

Results will be reported as the themes and subthemes identified after the thorough analysis and coding of the transcribed text material and will be accompanied by citations representing relevant themes and subthemes. Results are planned to be provided before the end of 2023.

**Conclusions:**

This study will likely provide new insights into user perspectives on a multi-operator self-exclusion service that involves both web- and land-based gambling operators, and which according to previous literature attracts many gamblers but also appears to have limitations and challenges in the target group of individuals with gambling problems. Policy and legislation implications, as well as clinical implications for treatment providers, will be discussed. Results and conclusions will be disseminated to policy makers in Sweden and internationally, as well as to peer organizations, treatment providers, and the research community.

**Trial Registration:**

ClinicalTrials.gov NCT05693155; https://clinicaltrials.gov/study/NCT05693155

**International Registered Report Identifier (IRRID):**

PRR1-10.2196/47528

## Introduction

Gambling disorder (GD) is an addictive condition known to cause overindebtedness, mental health consequences, and psychosocial problems in affected individuals and their concerned significant others. GD is the first addictive condition not involving a substance, and it is defined both in the diagnostic manual of the American Psychiatric Association, the DSM-5 (the *Diagnostic and Statistical Manual of Mental Disorders*, Fifth Edition) [1], and in the International Classification of Diseases (ICD) manual of the World Health Organization [2]. GD typically involves problematic gambling on either chance-based gambling modalities such as land- or web-based casinos, bingo or lotteries, sports or horse race betting, and poker or other card gambling. Severe consequences for family relationships are common [3], and psychiatric comorbidity is very common in individuals suffering from GD [4]. Likewise, GD is associated with overindebtedness [5] and with an increased risk of suicidal behavior including completed suicide [6,7]. GD can be treated using a psychotherapeutic approach, typically involving cognitive behavioral therapy, or through brief or more extensive motivational interventions or normative feedback interventions. Furthermore, pharmacological treatment strategies are applied, most commonly involving opioid antagonists. In addition, psychiatric comorbidities are fundamental to identify and treat [3]. However, treatment-seeking is typically low, and many patients with GD experience barriers against seeking treatment [8].

Voluntary self-exclusion from gambling venues or from web-based gambling sites is one preventive or harm-reducing strategy often applied. The concept behind this is the desire of individuals with gambling problems to control or discontinue their gambling behavior, that is, the voluntary choice to apply external control on gambling, in individuals who perceive a lack of control in their own behavior. Voluntary self-exclusion from gambling can be chosen by individuals regardless of whether they seek treatment or not. In addition, although people who choose self-exclusion typically may experience a gambling problem that they wish to control, self-exclusion may theoretically also be chosen by individuals without gambling problems. Previous data have revealed that individuals who choose to self-exclude have—in most but not all cases—gambling problems [9]. Thus, it is theoretically possible, although hitherto poorly demonstrated, that individuals without any gambling problems may wish to prevent themselves from developing potential gambling problems.

In many cases, self-exclusion services involve only one or a few land-based gambling venues or involve one or a few web pages of web-based gambling operators. Thus, a typical scenario is that a person with a GD chooses to self-exclude from that individual’s most common gambling sites or as many sites as possible but may relapse into gambling on other gambling sites. One part of this challenge is the fact that self-exclusion services are often operator-based and therefore involve only the gambling services of a specific gambling operator. Thus, breaching one’s own voluntary self-exclusion historically has been a challenge strongly limiting the efficacy of this harm reduction tool [10-13]. The highly diverse designs of such programs across countries and settings have been pointed out as a major limitation of this type of harm-reducing intervention [14]. In recent years, more transparent self-exclusion programs, easier to access, have been called for [15].

Based on these limitations, a new type of self-exclusion service, the Spelpaus service (literally “gambling break”) was introduced in Sweden in 2019, as part of the introduction of a new gambling market. The new gambling legislation allowed for a large number of gambling operators, previously often operating over the internet from overseas settings, to obtain a license to operate in Sweden, provided they adhere to this national self-exclusion system and other responsible gambling practices [16]. Spelpaus is a unique self-exclusion service, as it (1) is nationwide; (2) involves all licensed gambling operators, both web- and land-based (currently around 80 different operators are licensed to operate in Sweden); and (3) is independent of the operators themselves, administered by a governmental institution and therefore possible to access without any contact with gambling operators.

The actual effects of the Spelpaus service have not been assessed in research. However, it has been documented that Spelpaus attracts a large number of people; around 95,000 people on a given day are reported to be self-excluded from gambling through this system [17]. Thereby, the number of people self-excluded through this service clearly surpasses the number of people believed to experience a GD at a given time (which may, despite discrepancies between methods of measure, reach a number in the order of 30,000) [18]. Thus, this service appears to attract many people [19,20], both in the general population and among people attending a GD treatment unit [21].

However, one experience of Spelpaus is the emerging data indicating that despite the multi-operator, nationwide nature of this service, breaching the self-exclusion appears to be common in individuals with a GD. This type of breaching typically involves access to overseas, unlicensed web-based gambling operators [21]. In one web-based survey study, 38 percent of self-excluded individuals reported that they had continued to gamble during the self-exclusion period, and web-based casinos were the most common gambling type reported by individuals who relapsed in that context [22]. Furthermore, in patients with a GD, gambling under another person’s identity has been reported as a different way of breaching one’s self-exclusion [21].

Thus, Spelpaus appears to be a potentially effective harm reduction tool in order to prevent gambling in patients with GD or in people at risk of such problems, but major challenges regarding gambling outside of the jurisdiction appear to potentially limit the value of this service. However, given the popularity of this service, and the need to optimize harm-reducing interventions against GD, there is reason to study the effects and limitations of the self-exclusion service further. Hitherto, the experience of this new, unique self-exclusion tool is primarily based on quantitative research [19-22], whereas qualitative research may provide deeper knowledge about the lived experience of Spelpaus self-exclusion in individuals affected. Furthermore, the experience of concerned significant others has been little understood so far, and there is reason to suspect that the relationship of gamblers with their concerned significant others may change if they self-exclude in order to discontinue a problematic gambling pattern. Theoretically, the negative experience of concerned significant others from the gambling of their loved ones may be partly relieved if an external barrier can prevent the gambler from gambling further. In line with this, previous research has demonstrated that the choice of a person to self-exclude often involves the communication of this decision to the person’s loved ones, or sometimes is even part of measures required by the family [9].

The study aims to describe the experience of the Spelpaus service in gamblers, in order to deepen the understanding of the utility and challenges of this harm reduction tool. The study will use a semistructured interview design, in order to obtain a deeper knowledge than can be achieved in quantitative survey studies.

## Methods

### Study Procedure

This is a semistructured, qualitative interview study including individuals who gamble and who have an experience of Spelpaus self-exclusion. Interviews will be carried out primarily over the internet (or in exceptional cases face-to-face). Interviews will be recorded and transcribed verbatim, and the text will be analyzed using a content analysis on a descriptive level [23]. Interviews will be carried out by the second author of this paper, who has extensive clinical experience in psychiatric nursing and experience in qualitative research interviewing. The interview structure has been tested, and thereafter adapted (Textbox 1), in a pilot interview conducted with a respondent with a history of gambling problems and involved in peer support of patients with GD.

**Semistructured interview guide. The interview guide is meant to guide the qualitative interview, and therefore, the conversation is likely to move away slightly from the exact wording of the questions, based on the responses and associations made by the interviewee. Closed-ended questions will be asked as if they introduce a potential topic into the interview, and will not be handled, in the interview situation, as dichotomous quantitative study items.**

**General introduction to one another, introduction to the topic of the research, etc.**

- How old are you?
- Your gender (male, female, or other)?
- Your marital status (married, cohabitating, partner, or unmarried living alone)?
- What is your occupation?
- Which gambling types has/have been problematic for you? On how many gambling operators?
- Have you ever been in treatment for gambling problems? Which type of treatment?
- Have you ever had any problems related to the misuse of alcohol or drugs?
- Have you ever been in treatment (therapy or medication) for any kind of poor mental health?
- Tell me a little bit about your gambling problem. Why did you start gambling, and for how long have you had a problem? Which types of gambling have been involved, and on how many operators? Have you ever sought treatment, now or before? What type?
- How has your gamble influenced your concerned significant others (CSOs)?
- Tell me about your choice to self-exclude through Spelpaus? Which factors led to that?
- Did any of your CSOs have any influence on your decision to self-exclude?
- Do you have previous experience of other self-exclusion from gambling, before Spelpaus existed?
- How many times have you self-excluded, and for how long periods? Why did you choose those periods of time? Are you self-excluded right now, and for what duration?
- Tell me about your experience of Spelpaus. What has it meant to you, and to your CSOs? Do you believe it changed something for you? Did it reduce the inflow of gambling advertising, and how did that affect you? Which factors of Spelpaus have been favorable, and unfavorable, respectively?
- If you have received treatment for gambling problems, how has the Spelpaus self-exclusion affected that?
- Has your relationship with other people changed after your self-exclusion? How?
- Have you gambled during the Spelpaus self-exclusion? If no, which factors prevented you from that?
- After how long time of self-exclusion did you relapse into gambling? In what gambling types? Which were the factors in life that led to this gambling or which made it more difficult? In what way?
- Were you able to tell people that you had relapsed into gambling? How did that affect your relationship with these people?
- What’s your general impression of Spelpaus? What works and what does not? What could be improved in this system?
- Is there anything you feel we have forgotten to ask, and that you would like to add when it comes to your own and your CSO’s impressions of Spelpaus?

The analysis will be a content analysis, more specifically involving a text analysis of transcribed text, in order to interpret and provide an understanding of the narrative of the study participants. The analysis will be conducted according to the following sequence: (1) repeated reading of the entire transcribed text material, (2) coding of meaning units, (3) categorization of identified themes and subthemes, (4) provision of citations describing the categories, (5) calculation of the number of times a theme appears in the transcribed material, (6) comparison of interviews and identification of differences and similarities between them, and (7) the search for explanations to such differences. Analyses will be carried out by the second and third authors of this paper. The analytic procedure and discussions within the study group will aim to optimize trustworthiness in the analyses [24].

### Study Questions

What is the lived experience of users who have been self-excluded through the Spelpaus service? Which have been the major advantages and the major disadvantages of self-exclusion in gamblers, and in which way has this been affected by the possibility of breaching one’s self-exclusion through gambling on overseas gambling sites? Which potential improvements can be made to the Spelpaus self-exclusion service, according to users? What is the lived experience of gamblers who have self-excluded at Spelpaus, regarding how this has changed their relationship with their concerned significant others?

### Study Participants

Study participants will be recruited through purposeful sampling. Individuals will be recruited because they are either (1) individuals recruited from social media advertising and who have a gambling problem and experience of the Spelpaus self-exclusion service or (2) patients with a GD at the Gambling Disorder Unit of Region Skåne and who have experience of the Spelpaus self-exclusion service. Participants will primarily be recruited through social media recruitment. In case of a slow recruitment procedure, patients at the treatment unit will also be addressed with a question about their interest in taking part in the study. Participants receive a smaller economic compensation, in the format of cinema ticket gift cards. The study aims to recruit a sufficient number of gamblers for saturation to be obtained in the data material. This will include the aim to include participants with a wide variety of experiences concerning the Spelpaus service, including different reasons for self-excluding, and involving both women and men.

Saturation will be considered through a continuous analytic procedure, where collected data material is compared to previously collected data. Thus, no exact number of expected interviews can be decided and reported beforehand, and given the continuous process of assessing saturation, this may require the research group to add additional participants during ongoing analysis. However, based on our experience from previous studies, and based on the intention to assess potentially diverse experience and potentially experience in women and men, it has been reported in the ethics application that the final number of included clients may reach 20. Thus, despite this uncertainty, we expect the final number of participants to be in the range of 12-20.

### Setting

The outpatient Gambling Disorder Unit in Region Skåne, Sweden, from which a minority of participants may be recruited, is one of very few units treating GD in a health care setting in Sweden, and has been described previously [21,25]. The unit is part of the Region Skåne Competence Center Addiction, which is a research and development unit closely related to an addiction medicine research group. This treatment unit welcomes patients aged 18 years and older, who experience gambling problems (typically diagnosed with GD [25]) and who live in the Skåne county, a region with a population of 1.4 million inhabitants. The facility is located in the city of Malmö, which is the most urban center of the region.

### Ethical Considerations

The study has been approved by the Swedish Ethical Review Authority (overall project approved, file number 2022/06933-01, and amendment number 2023/01684-02 regarding change of interview guide and social media route of recruitment).

### Study Preregistration

The study has been preregistered at ClinicalTrials.gov (NCT05693155).

## Results

Results from this study are scheduled to be presented before the end of 2023. Findings will be reported as themes and subthemes, which will be identified after transcribing and coding the text material. Results will be accompanied by citations which will be selected to represent relevant themes and subthemes.

## Discussion

### Principal Findings

This study will be the first to examine, in a qualitative in-depth research design, the experiences of the present self-exclusion system, which is novel and relatively unique in international comparison. Given the novelty of the service, the study may have the potential to guide improvements in this self-exclusion service and the regulations around it.

Findings from this study will be disseminated both within the research community and to peer organizations addressing individuals with GD and their concerned significant others. In addition, findings will be discussed formally and informally with policymakers in government settings, public authorities, and with treatment providers and organizations involved in the prevention and early detection of addictive disorders. Furthermore, findings from this qualitative study are likely to inspire upcoming quantitative studies that can quantify and follow, over time, key aspects of gambling self-exclusion identified through this research.

### Strengths

The possibility to address more detailed aspects of different reasons for self-excluding, other than what can be obtained through closed-ended quantitative study items, is one advantage of the qualitative study design. Another strength of this study is that it is the first to assess the present type of nationwide, multi-operator system which involves both land-based, traditional gambling types, and web-based gambling sites. In addition, it is conducted in a situation where web-based gambling, either within or without the jurisdiction in which the system is introduced, is likely a larger challenge than ever. Therefore, this study has the potential to add updated information about a novel type of system that meets challenges that have been less significant in older similar systems.

### Limitations

Limitations of the study may be related to the recruitment procedure. As it is hitherto largely unknown which features may characterize individuals who self-exclude from the present type of self-exclusion service, other than people with manifest gambling problems, it is a challenge to reach the full variety of reasons for self-excluding or types of self-exclusion–related experiences. Therefore, wide social media recruitment has been chosen, although patients with a known GD at a treatment facility may also be added, in case of a slow recruitment procedure through the open social media channel. Thus, given the lack of knowledge so far, we have chosen a wide recruitment method but still, the inclusion of participants may potentially be biased by features of social media users and users who are specifically attracted by this type of study advertising. These aspects will be of importance to discuss in the analyses and the results reported in upcoming publications.

## Abbreviations

DSM-5: Diagnostic and Statistical Manual of Mental Disorders, Fifth Edition
GD: gambling disorder
ICD: International Classification of Diseases

## Data Availability

The data sets generated or analyzed during this study are not publicly available due to requirements of confidentiality in line with the present ethics permission and can be made available from the corresponding author on reasonable request, in an aggregated nonidentified form, and after review by the ethics authority.
